# Angular dose dependency of MatriXX TM and its calibration

**DOI:** 10.1120/jacmp.v11i1.3057

**Published:** 2010-01-28

**Authors:** Luciant D. Wolfsberger, Matthew Wagar, Paige Nitsch, Mandar S. Bhagwat, Piotr Zygmanski

**Affiliations:** ^1^ Division of Medical Physics and Biophysics Department of Radiation Oncology Dana‐Farber Cancer Institute/ Brigham and Women's Hospital Harvard Medical School Boston MA USA

**Keywords:** MatriXX, angular dependence, calibration

## Abstract

One of the applications of MatriXX (IBA Dosimetry) is experimental verification of dose for IMRT, VMAT, and tomotherapy. For cumulative plan verification, dose is delivered for all the treatment gantry angles to a stationary detector. Experimental calibration of MatriXX detector recommended by the manufacturer involves only AP calibration fields and does not address angular dependency of MatriXX. Angular dependency may introduce dose bias in cumulative plan verification if not corrected. For this reason, we characterized angular dependency of MatriXX and developed a method for its calibration. We found relatively large discrepancies in responses to posterior vs. anterior fields for four MatriXX (Evolution series) detectors (up to 11%), and relatively large variability of responses as a function of gantry angle in the gantry angle ranges of 91°–110° and 269°–260°. With our calibration method, the bias due to angular dependency is effectively removed in experimental verification of IMRT and VMAT plans.

PACS number: 87.56Fc

## I. INTRODUCTION

MatriXX (IBA Dosimetry, Bartlett, TN) is a 2D detector developed for megavoltage dosimetry.^(^ ^1^ ^–^ ^4^ ^)^ One of its potential applications is experimental verification of patient specific IMRT (and more recently VMAT) plans. Patient specific dose verification can be carried out with MatriXX either mounted on the rotating gantry or positioned on the treatment couch. In the latter case, cumulative plan dose in a single plane can be measured. The inherent buildup and backscatter material of the detector is equivalent to about 0.3 cm and 3.5 cm of water, respectively. Calibration of MatriXX includes correction of gain of individual ionization chambers ($k_{\text{gain}}$ factor) and absolute calibration of the detector response ($k_{\text{user}}$ factor). The calibration of gain of individual detectors ($k_{\text{gain}}$ factor) is performed by the manufacturer and verified with large size open beams by the user to be acceptable. The user has to experimentally determine $k_{\text{user}}$ factor, which converts the charge collected by the internal electrometer of MatriXX to the dose deposited in the detector plane at a given calibration depth and field size (selected by the user). As a result of $k_{\text{user}}$ calibration, the dose measured by MatriXX is absolute.

Absolute calibration ($k_{\text{user}}$ factor) of the detector recommended by the manufacturer is performed solely for AP calibration fields with the assumption that the inherent buildup is 0.3 cm plus the additional buildup selected by the user (e.g. 0.3cm+depth of additional solid water). To the best of our knowledge, angular dependency of MatriXX and its calibration for other than AP fields (PA, posterior oblique, and lateral fields) have not been recognized as required and have not been reported. We have tested several MatriXX Evolution detectors and observed rather large dose bias (up to 8%–11%) for the posterior beams and only relatively small uncertainty of 1% for the anterior beams. This sort of asymmetric detector response cannot be accounted for neither by the uncertainties in the density of the multilayer materials inside the detector nor by the uncertainties in the Hounsfield units (HU) in the planning CT. For these reasons, we have determined angular dependency of MatriXX and developed a calibration procedure for all gantry angles.

## II. MATERIALS AND METHODS

### A. General

The goal of the calibration procedure described here is to determine and to correct the inherent angular dependency of MatriXX for megavoltage beams for the purpose of IMRT and VMAT QA. This inherent angular dependency of MatriXX is independent of attenuation introduced by a phantom used in the QA and thus is specific only to the MatriXX detector irrespective of the additional buildup/backscatter material provided by the user. Further, it is to be understood as the dependency of the whole device (ionization chambers inserted in a complex structure), and not as dependency of an individual ionization chamber abstracted from its immediate surrounding. The individual ionization chambers by themselves may not be sensitive to the directionality of the beam; however, when inserted in the multilayer structure with high‐Z materials and air, the response of the whole device may be.

**Figure 1:**
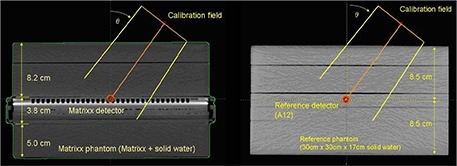
CT of MatriXX and reference phantoms. MatriXX phantom (left): MatriXX detector sandwiched between 8.2 cm (ant) and 5 cm (post) solid water slabs; reference phantom (right): A12 ionization chamber placed at the center of solid water phantom of total thickness of 17 cm.

In absolute calibration with the AP fields, there is an assumption that the dose to be measured in QA is not the actual dose deposited to the ion chambers themselves (dose to the walls, electrode, or gas filled cavity) but it is the dose in water at the same water equivalent depth. Accordingly, in the development of the calibration technique for the angular dependency, we assume that the signal recorded by the detector array is to be converted to a dose in water for similar depths and angles. Therefore, the angular calibration technique requires measurements in two types of phantoms. One of the phantoms is *the MatriXX phantom* consisting of MatriXX device and additional buildup/backscatter material as, for instance, depicted in the left panel of Fig. 1. The other phantom, which we will call *the reference phantom*, is a uniform phantom without the internal structure specific to the MatriXX device as, for instance, depicted in right panel of Fig. 1. The reference phantom is to match the geometry of the MatriXX phantom and its water equivalent depths to the point of measurement as closely as possible in the clinical scenario. In the angular calibration process, it is assumed that the doses measured in the MatriXX phantom are converted to the doses in the reference phantom. For that reason, when these two phantoms are used in the QA, the measurements of patient plans are to be performed in the MatriXX phantom and then compared to the calculations in the reference phantom for optimal results.

Further, it is assumed that the doses in the reference phantom are measured with an independent detector, which does not have any measurable angular dependency. We refer to this detector as the reference detector.

### B. Experimental setup

The specific measurements presented here are performed with four MatriXX (Evolution series) detectors, for brevity referred to as “MatriXX”, in 6 MV beams. The build‐up and scatter materials were 30cm×30cm solid water slabs (8.2 cm over the top and 5.0 cm underneath the MatriXX device vs. 8.5 cm and 8.5 cm in the reference phantom). The total water equivalent thickness of MatriXX phantom is thus 17 cm and was matching the thickness of the reference phantom. However, in the lateral direction, the water equivalent thickness of the MatriXX phantom does not exactly match the width of the reference phantom. For instance, the high‐Z plate and the ion chamber array composed partially of air cavities are the two structures that are not imitated in the reference phantom. More detailed matching of the internal structure of MatriXX would be difficult to achieve in the clinical setting and, in fact, is not necessary in the correction technique presented here. An A12 ionization chamber is used as a reference detector. A12 is not sensitive to the directionality of the beam when placed along the axis of gantry rotation.

In order to determine a correction factor CF as a function of gantry angle Θ open beam fields of 10cm×10cm size were irradiated for gantry angles $\Theta {=0}^{{}^{\circ}}{,10}^{{}^{\circ}},\ldots {,350}^{{}^{\circ}}$ (every 10°) and every 1° for lateral angles Θ=90°−110° and Θ=270°−260°. The CF(Θ) is a slowly varying function except at lateral angles. Doses were measured in cGy using “movie” data acquisition mode (dose images saved as “snaps” every 0.5 sec).

### C. Calibration factor

Let us establish that dose measured with MatriXX for given gantry angle Θ is D(Θ) and dose measured with a reference detector (ionization chamber placed at the same location in the reference phantom) is $D_{\text{ref}}$ (Θ). If there were no inherent angular dependency of the MatriXX, then $D(\Theta )/D_{\text{ref}}(\Theta )$ would equal 1 at all angles. In a symmetric phantom setup, ideally the dose measured for a given gantry angle D(Θ) should be equal to the dose for the opposing gantry angle $D\;(\Theta \;+\;180^{{}^{\circ}})$.

In reality, due to inherent angular dependency of MatriXX ionization chambers, these equalities are not fulfilled, as will be seen in the Results section. Thus, in order to calibrate MatriXX for other than AP gantry angles, we define a calibration (correction) factor:

(1) $$CF(\theta )=\frac{D\;(\theta )}{D_{\text{ref}}\;(\theta )}$$

The intended usage of calibration factor CF (Θ) is as follows. If dose measured with MatriXX for a field to be experimentally verified is $D_{\text{QA}}(\Theta )$, then the calibrated dose is:

(2) $$D_{QA}^{\text{cal}ib}(\theta )=\frac{D_{QA}(\theta )}{CF(\theta )}$$

Where, for optimal results, the determination of CF (Θ) and experimental verification of $D_{\text{QA}}(\Theta )$ and are performed using the same QA and calibration phantom geometry.

### D. Correction of MatriXX doses

Currently, the MatriXX software does not support angular calibration and for this reason we used an in‐house software, which takes as input MatriXX doses, corrects them using CF‐factor and exports the resulting doses back to MatriXX software for comparison with the treatment planning system.

The difference between AP‐type calibration ($k_{\text{user}}$ factor) and angular calibration as defined by Eqs. (1) and (2) is that in the angular calibration for each specific field has to be corrected depending on the gantry angle before the cumulative dose is calculated. For IMRT fields, the gantry angle is fixed for each field. However for VMAT, the gantry is moving. Thus the best way to correct the doses is to read each dose per frame and correlate it with the gantry angle to determine proper value for CF(Θ).

MatriXX records dose per unit time, called “snaps”. These “snaps” are exported to ASCII files. The in‐house software reads all exported “snaps” taken during the irradiation for each field and divides snaps by a correction factor CF(Θ) depending on the gantry angle. The gantry, gantry speed, dose rate information is taken from the plan by reading DICOM RT plan files. Alternatively, correlation of gantry angle with snap time can be done manually. For instance, in the treatment planning system Eclipse (Varian Medical Systems, Palo Alto, CA) the gantry speed and dose rate information are displayed in the “Details” of “MLC properties”. Based on this knowledge and on the knowledge of which snaps correspond to the onset/end of radiation, a unique correlation between the gantry angle and snap time is established. The onset/end of radiation is clearly observed as the change from zero to non‐zero signal in the snap images. Independently, the overall irradiation time is measured with a digital clock to verify that the integrated time (total arc length divided by gantry speed times snap time) is the same as the measured total time with the clock. We have not observed any measurable discrepancy between the measured and calculated times, so in practice this step could be avoided. For all cases considered, the gantry speed was constant and equal to 4.8°/sec. The correction was applied for each snap (every 0.5 sec or every 2.4°).

### E. Application to patient specific QA

We performed patient‐specific IMRT and VMAT (RapidArc, Varian Medical Systems, Palo Alto, CA) verification using MatriXX in a QA setup similar to calibration setup (Fig. 1). In addition, we repeated IMRT and VMAT QA with A12 and with EDR2 films in a similar setup. When QA was performed with MatriXX, we corrected the measured doses for each field using Eq. (2) and the in‐house software. The corrected dose distributions for individual fields were summed and compared with the cumulative doses from the planning system (Eclipse). Similarly, we compared doses measured with film/A12 with the TPS doses. A total of 3 IMRT and 2 VMAT patient‐specific QA were performed for this study (Table 1. IMRT plans consisted of $5H\;(255^{{}^{\circ}},\;315^{{}^{\circ}},\;45^{{}^{\circ}},\;105^{{}^{\circ}},\;180^{{}^{\circ}}),\;7C\;(205^{{}^{\circ}},\;260^{{}^{\circ}},\;310^{{}^{\circ}},\;0^{{}^{\circ}},\;50^{{}^{\circ}},\;100^{{}^{\circ}},\;155^{{}^{\circ}})$ and $5Y\;(185^{{}^{\circ}},\;310^{{}^{\circ}},\;350^{{}^{\circ}},\;25^{{}^{\circ}},\;145^{{}^{\circ}})$ fields, and VMAT plans consisted of 2 full arcs $\left(179^{{}^{\circ}}\;\to \;180^{{}^{\circ}}\text{and}\;180^{{}^{\circ}}\to \;179^{{}^{\circ}}\right)$ for each plan.

**Table 1:** Treatment plan parameters.

*Plan Modality*	*Gantry Angle*	*Energy*
IMRT 5H	255°, 315°, 45°, 105°, 180°	6 MV
IMRT 7C	205°, 260°, 310°, 0°, 50°, 100°, 155°	6 MV
IMRT 5Y	205°, 260°, 310°, 0°, 50°, 100°, 155°	6 MV
VMAT 1	2 full arcs $\left(179^{{}^{\circ}}\;\to \;181^{{}^{\circ}}\text{and}\;181^{{}^{\circ}}\to \;179^{{}^{\circ}}\right)$	6 MV
VMAT 2	2 full arcs $\left(179^{{}^{\circ}}\;\to \;181^{{}^{\circ}}\text{and}\;181^{{}^{\circ}}\to \;179^{{}^{\circ}}\right)$	6 MV

In order to correct MatriXX doses, we had to export the raw doses from MatriXX software (OmniPro IMRT, Omnipro Systems Inc., San Francisco, CA). After correction of data, the results were imported back to MatriXX software for further analysis.

Treatment planning system was commissioned using standard methodology.^(5)^ MLC parameters inside planning system were the same for IMRT and VMAT plans.

### F. Other issues

#### F. 1 Water equivalent buildup/scatter of MatriXX

To verify that the total water equivalent thickness of MatriXX is the same as stated by the manufacturer (3.8cm=0.3cm buildup +3.5cm backscatter), we placed MatriXX as in Fig. 1 but varied the total thickness of the phantom simulating the TPR setup. The MatriXX phantom was placed on additional slabs containing A12 ionization chamber. We measured attenuation signal due to MatriXX phantom for variable total thickness and compared it with a similar signal measured solely with solid water. A12 was placed in isocentric setup.

#### F. 2 High‐Z material and HU units

Finally, we calculated doses for open AP/PA fields with and without corrections for phantoms in Fig. 1 using the planning system to examine whether inhomogeneity correction based on path length correction (in AAA dose calculation algorithm) can account for dose discrepancy corresponding to MatriXX doses. We calculated doses for the MatriXX CT with the original and altered Hounsfield units (HU). HU were modified in the planning system, to verify if inaccurate HU (due to the bias in the planning CT reconstruction) could, in principle, be the cause of the large AP/PA dose discrepancy. We created a contour surrounding the high‐Z materials inside MatriXX CT and ascribed to them an artificially high HU value until we matched the measured doses measurement for PA fields (for anterior fields, the TPS doses were within 1% from MatriXX; it is only for posterior fields that the large dose discrepancies exist).

#### F. 3 CF(Θ) for PA vs. lateral fields

In a further attempt to determine the cause of the inherent angular dependency of MatriXX for AP/PA fields compared to the lateral fields we calculated water equivalent depths to the isocenter (Fig. 1) as a function of gantry angles using the planning system (Eclipse) and correlated them with similar dose values. The high‐Z region was of special interest, as was its influence on the lateral fields vs. posterior fields.

#### F. 4 Off‐axis dependence

In the above angular calibration procedure, there is an underlying assumption that the angular response of all ionization chambers in MatriXX array is similar and, therefore, that the correction factor is shift invariant within the detector plane. To verify the angular dependency for the ion chambers at off‐axis locations within the detector array we compared measured doses for open beams at off‐axis locations. As a reference dose, we used the dose profiles collected in a water tank with small‐volume ion chamber (I10).

## III. RESULTS

### A. Angular dependency

**Figure 2:**
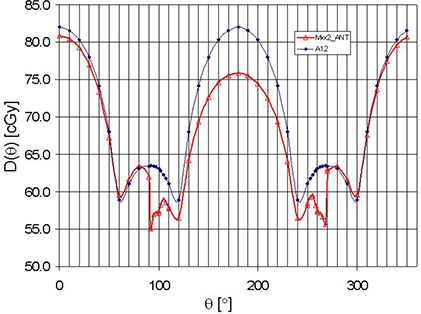
Doses as a function of gantry angle in MatriXX and reference phantoms. The attenuation due to phantom shape is naturally included in these profiles.

**Figure 3:**
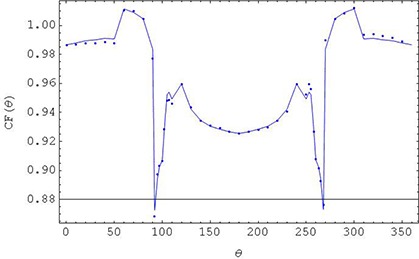
Angular dependency of one of the MatriXX detectors. Data points are the measured doses relative to A12. Solid line is a cubic interpolation of mirrored data $\text{CF}\;(\Theta )\;\to \;(\text{CF}(\Theta )\;+\;\text{CF}(360^{{}^{\circ}}\;-\;\Theta )\;/\;2$.

Doses as a function of gantry angle measured with one of the four MatriXX detectors and the A12 ionization chamber are shown in Fig. 2. These doses reveal the total effect due to the inherent angular dependency and the attenuation (in turn, due to rectangular phantom used in the QA setup). Figure 3 shows just the inherent angular dependency of MatriXX. Angular dependency of other MatriXX detectors was similar in shape but slightly different in magnitude. For instance, AP to PA dose ratio for the four MatriXX detectors ranged from about 7% to 11%. We measured some of the data multiple times and found good reproducibility of data points and, therefore, stability of MatriXX response to within 0.5%−1%. Absolute dose for AP fields was also found to be within 1% consistent with $k_{\text{user}}$ calibration (100 cGy = 100 MU, for reference field size and depth). Slight deviations between MatriXX and A12 for AP fields arise from the inherent uncertainties of both of these detectors. Asymmetry of the profiles observed for MatriXX (for A12 there was practically no asymmetry) with respect to 180° gantry angle may be due to difference of the inherent structure and due to setup, which is more sensitive to misalignment than for A12.

### B. Patient‐specific QA

**Figure 4:**
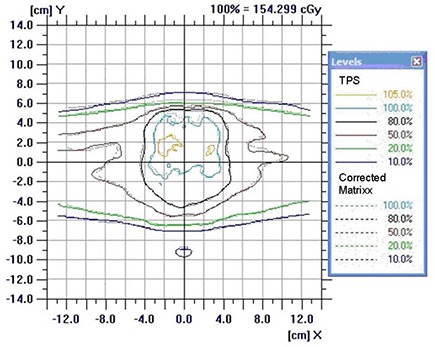
Typical dose profiles for cumulative IMRT and VMAT plans measured with MatriXX (corrected for angular dependency with CF approx./equal to 0.97) and compared to TPS. Before correction there was a dose bias of about 3%, and the isodose lines did not match well.

Patient‐specific IMRT and VMAT QAs performed with MatriXX as recommended by the manufacturer (not corrected for angular dependency) show dose distributions similar in shape to those calculated by the treatment planning system (TPS). However, the magnitude of the uncorrected MatriXX doses is consistently smaller than the corresponding magnitude of TPS doses (Table 2, column 1). After correction of MatriXX doses with the CF(Θ) factor, the agreement between measurement and TPS is improved (Table 2, column 2) and isodoses match (Fig. 4). Average (over all gantry angles used in the plan) correction factors for each plan are shown in Table 2, column 3. The A12 ionization chamber doses with respect to TPS doses in the corresponding reference phantom are displayed in Table 2, column 4.

**Table 2:** Average relative doses $D_{1}{/D}_{2}$ within PTV for patient specific QA.^a^

$D_{1}$	*MATRIXX NOT CORRECTED*	*MATRIXX CORRECTED*	*MATRIXX NOT CORRECTED*	*A12 IN REFERENCE PHANTOM*
$D_{2}$	*TPS in MatriXX Phantom*	*TPS in Reference Phantom*	*MatriXX Corrected*	*Reference Phantom*
IMRT 1 5H	97.3%	99.3%	95.6%	99.3%
IMRT 2 2C	97.4%	98.3%	94.8%	98.6%
IMRT 3 3Y	97.5%	100.1%	96.6%	98.6%
VMAT 1	97.0%	100.7%	96.5%	100.9%
VMAT 2	98%	101.4%	97.1%	99.5%

^a^ Average relative doses $D_{1}/D_{2}$ within PTV for patient specific QA performed with various methods: uncorrected MatriXX dose divided by planning system dose in MatriXX phantom, corrected MatriXX dose divided by planning system dose in reference phantom, and uncorrected MatriXX dose divided by corrected MatriXX dose, A12 dose divided by planning system dose in reference phantom. MatriXX doses are measured in the MatriXX phantom and A12 doses in the reference phantom

It needs to be stressed that all the measurements and TPS calculations have intrinsic uncertainties (noise and bias) common to the standard QA techniques used and, therefore, to the corrected data as well. Thus attention needs to be paid to a trend in this comparison rather than specific numbers for a given plan. For instance, IMRT3 and VMAT2 were plans with higher dose gradients within PTV; thus, this may be reflected in a larger difference between A12 and MatriXX measurements. IMRT2 shows consistent lower dose in 5 measurements compared to TPS. Further, it needs to be pointed out that according to Fig. 4, TPS water equivalent path length corrections for ranges $\Theta \;=\;91^{{}^{\circ}}-110^{{}^{\circ}}$ and $\Theta \;=\;269^{{}^{\circ}}-260^{{}^{\circ}}$ partially account for angular dependency of MatriXX in these gantry angle ranges. However for angles $\Theta \;=\;120^{{}^{\circ}}-250^{{}^{\circ}}$, TPS dose calculation fails to account for the effect seen in Fig. 3. These two effects are combined in the cumulative plan verification. Based on the limited cases presented, a dose bias of up to about (−3%) can be observed in experimental verification of IMRT and VMAT plans if not corrected for angular dependency of MatriXX.

### C. Other issues

Dose attenuation as a function of varying total phantom thickness (TPR measurement) for MatriXX phantom vs. solid water phantom measured with A12 was within 1%, confirming that water equivalent thickness of MatriXX is as stated by the manufacturer (total of 3.8 cm) for all practical purposes (QA in MV beams). Thus this confirms that the MatriXX and reference phantoms have exactly matching water equivalent depths for AP and PA fields.

**Figure 5:**
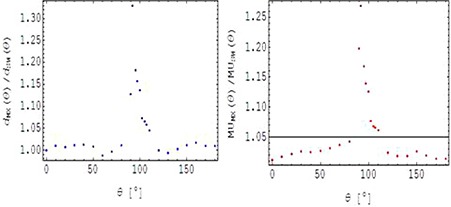
Relative water equivalent path length (left) and relative monitor units (right) as functions of gantry angle calculated in planning system. Relative is defined for MatriXX phantom with respect to the reference phantom. Monitor units are for the same amount of dose at the isocenter for 10×10cm open beam.

Water equivalent path length as a function of gantry angle calculated from CT using treatment planning system is shown in Fig. 5. It should be noted that the isocenter for the purpose of path length calculation was selected in the plane just between the ionization chamber and the adjacent 3 mm buildup material. As a result, this may not be the effective point of measurement of the MatriXX ionization chambers. Further, the calculation of path length in the planning system depends on the contouring of the “body” structure and has intrinsic uncertainties. In the case of AP and PA fields, notably, the effective path lengths for AP and PA fields are within about 1mm from the 8.5 cm. The difference between the true effective path length inside MatriXX (confirmed by the aforementioned measurements with A12) and the path length calculated by the planning system is ascribed to these uncertainties and a potential different usage of “path length” in the planning system. We did not attempt to characterize the latter uncertainties. In the right panel of Fig. 5, the ratio of water equivalent path length to physical path length is uniform (within about 2%) for most gantry angles. However, as the gantry crosses $\Theta \;=\;90^{{}^{\circ}}$ towards posterior angles, there is a sharp increase in the relative effective path length. The maximum occurs about $\Theta \;=\;95^{{}^{\circ}}$ and then the relative effective path length decreases, albeit less abruptly. The cause of this sharp peak in the path length profile is apparently the high density (high‐Z material=0.5cm thick bright region just below the ionization chambers in Fig. 1) along the path lengths for angles $\Theta \;=\;91^{{}^{\circ}}-110^{{}^{\circ}}$. Findings are similar for gantry range $\Theta \;=\;269^{{}^{\circ}}-260^{{}^{\circ}}$. This thus proves that the planning system properly takes into account the attenuation due to high‐Z materials for all angles.

**Figure 6:**
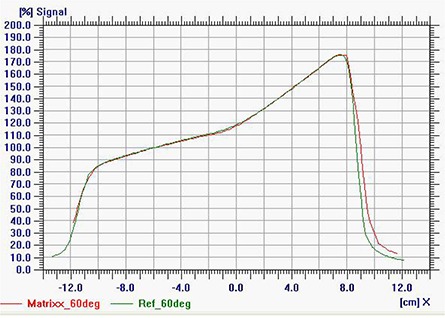
Dose profiles measured with MatriXX and reference ion chamber in water for 10×10cm at gantry angle 60°.

Correction factor CF was found to be shift‐invariant, meaning that the response of the ion chambers in the MatriXX array was the same irrespective of the location. Figure 6 shows an example of dose profiles measured with MatriXX and reference detector (small ionization chamber) for 10cm×10cm anterior oblique field $\left(\Theta \;=\;60^{{}^{\circ}}\right)$. Similar dose profiles for other anterior and posterior oblique angles show that rescaling the MatriXX doses by CF leads to a relatively good agreement (within about ± 0.7%) for all ion chamber locations. The larger discrepancies at the edges of the fields are most likely due to the uncertainty in the gantry angle and jaw settings, and due to the averaging effect introduced by MatriXX detectors which are about 4 mm in diameter, 5 mm in depth and are spaced every about 7 mm.

## IV. DISCUSSION

### A. Causes of inherent angular dependency

The rather large discrepancy in MatriXX response to PA vs. AP beams (up to 11%) cannot be ascribed purely to the uncertainties in water equivalent thickness (calculation of attenuation for the inhomogeneous MatriXX structure in the planning system). There are several reasons for that:

- Direct measurements confirm the value of water equivalent thickness of MatriXX stated by the manufacturer (0.3cmbuildup+3.5cm backscatter=3.8cm total).
- Angular dependency profile CF(Θ) in the range $\Theta \;=\;120^{{}^{\circ}}-250^{{}^{\circ}}$ has a minimum for $\Theta \;=\;180^{{}^{\circ}}$ and is closer to 1 for the adjacent angles. If attenuation were responsible for dose differences between AP/PA fields, CF(Θ) would be deviating more from 1 for the adjacent angles than for the PA field.
- Effective path length calculation in the planning system is consistent with the 8.5 cm value, and thus uncertainties in path length calculation cannot be responsible for 11% dose difference. In order to obtain an agreement between TPS and MatriXX measurements for PA vs. AP beams, one would have to increase the HU for 3.5 cm backscatter to about HU = 3000, which is extremely unphysical and which would cause problems for angles other than 180°.
- Calculation of dose for AP/PA fields in the planning system does not depend on whether the inhomogeneity corrections are used or not in the dose calculation model (within 1%)

Thus we conclude that the difference in dose for PA vs. AP fields is mostly due to some other effects occurring at the air‐high–Z material interface for the PA beams, and not to the overall path length. We also conclude that these dose discrepancies cannot be easily “corrected” or accounted for inside the planning system in a consistent fashion, and a correction of the measured doses is required. We have shown that such a correction is feasible and is cleaner than adjusting various factors inside the planning system.

On the other hand, in the case of lateral beams in the ranges of $\Theta \;=\;91^{{}^{\circ}}-110^{{}^{\circ}}$ and $\Theta \;=\;260^{{}^{\circ}}-269^{{}^{\circ}}$, we can conclude that the effective path length is a significant contributor to the lower dose for these angles (a sudden dose drop from 96% to 88%, Fig. 3). Thus if only these angles would be considered in QA, and if calculation of doses in the planning system would be done in the MatriXX phantom with inhomogeneity correction turned on, the dose agreement with the measurement would be satisfactory (considering that determination of dose is very sensitive to the gantry angle in this region – see Figs. 2–4).

### B. Calibration method

Based on the limited cases presented, a dose bias of not more than about (−3%) can be observed in experimental verification of IMRT and VMAT plans if not corrected for angular dependency of MatriXX. In our routine QA, we have calculated the correction CF for more than a hundred plans and we find that the dose correction may reach values up to 4%, but is typically about 3%. Whether this bias appears small or large is to be judged by individual user of QA equipment. If, for instance, the goal of QA is to achieve about 1% accuracy, then this study shows how to approach this goal in a systematic fashion rather than adjusting TPS parameters influencing the dose calculation.

Calibration of angular dependency for IMRT or VMAT based on CF(Θ) reveals improved (and satisfactory) QA results. However, it needs to be noted that calibration of IMRT or VMAT fields in the gantry angles $\Theta \;=\;91^{{}^{\circ}}-110^{{}^{\circ}}$ and $\Theta \;=\;260^{{}^{\circ}}-269^{{}^{\circ}}$ may be quite sensitive to misalignments (AP/PS shifts and gantry rotations), because of high gradient in CF(Θ) for these gantry ranges. Further, this calibration method focuses on correction of angular dependency based on the measurement of the angular dependency of the central ionization chambers only. There may be slightly different dependency of other ionization chambers in the MatriXX array for angles $\Theta \;=\;91^{{}^{\circ}}-110^{{}^{\circ}}$ and $\Theta \;=\;260^{{}^{\circ}}-269^{{}^{\circ}}$ gantry ranges. Fortunately, the total range of these angles is relatively small and, at least in the presented cases, they do not seem to greatly bias the overall IMRT or VMAT QA.

The correction method was developed to correct up to 8%–11% bias which was observed for some gantry angles (PA fields), and to decrease the overall uncertainty in the cumulative plan measurement from about 3% to the level of about 1%. We believe that decreasing the uncertainties below 1% would be very difficult to achieve with MatriXX due the aforementioned complications.

The calibration factor CF(Θ) is asymmetric, but for the purpose of the correction of QA doses, it is possible to derive a symmetric profile as seen in Fig. 2.

We also point out that there is a potential hidden danger in using MatriXX without proper identification of sources or errors and consistent correction of the angular dependency. For instance, after finding dose discrepancies between the planning system and those measured with MatriXX, one may be tempted to (incorrectly) adjust the MLC parameters (or other adjustable factors) inside the planning system and thus force a better agreement.

## V. CONCLUSIONS

We show that the rather large dose discrepancy between AP and PA fields measured with MatriXX Evolution cannot be accounted for by water equivalent path length corrections in the planning system and is most likely due to effects occurring at the air‐high–Z material interfaces. With the proposed correction method it is possible to decrease the uncertainties in the QA process down to 1% level.

## ACKNOWLEDGEMENTS

We would like to express our thanks to several people at IBA group for providing technical information, and to Laurence Court and Dan Cail for their clinical insights.

## Supporting information

Supplementary Material Files
