# Supplementary figures and images for: Angular dose dependency of MatriXX TM and its calibration
Source: J Appl Clin Med Phys. 2010 Jan 28;11(1):241–51. doi: 10.1120/jacmp.v11i1.3057 (PMC5719776; doi:10.1120/jacmp.v11i1.3057)

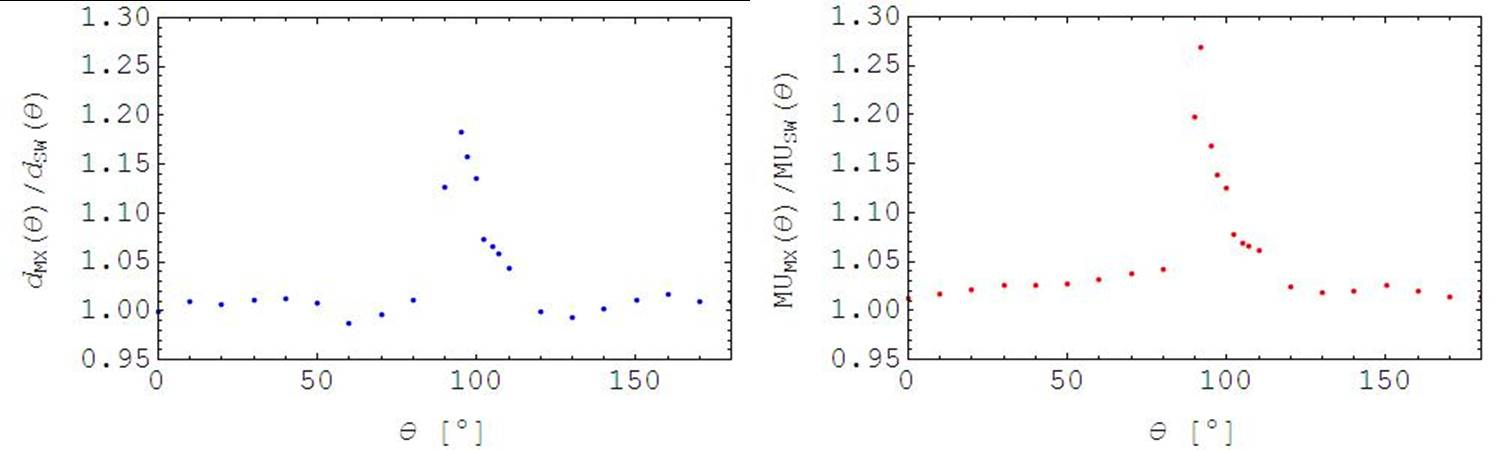

Supplement: Supplementary file 1 — Supplementary Material Files [file ACM2-11-241-s001.jpg]
